# *Mucuna pruriens* and Its Major Constituent L-DOPA Recover Spermatogenic Loss by Combating ROS, Loss of Mitochondrial Membrane Potential and Apoptosis

**DOI:** 10.1371/journal.pone.0054655

**Published:** 2013-01-22

**Authors:** Akhand Pratap Singh, Saumya Sarkar, Muktanand Tripathi, Singh Rajender

**Affiliations:** Division of Endocrinology, Council for Scientific and Industrial Research-Central Drug Research Institute, Lucknow, India; Massachusetts Eye & Ear Infirmary, Harvard Medical School, United States of America

## Abstract

**Background:**

The Ayurvedic medicinal system claims *Mucuna pruriens* (MP) to possess pro-male fertility, aphrodisiac and adaptogenic properties. Some scientific evidence also supports its pro-male fertility properties; however, the mechanism of its action is not yet clear. The present study aimed at demonstrating spermatogenic restorative efficacy of MP and its major constituent L-DOPA (LD), and finding the possible mechanism of action thereof in a rat model.

**Methodology/Findings:**

Ethinyl estradiol (EE) was administered at a rate of 3 mg/kg body weight (BW)/day for a period of 14 days to generate a rat model with compromised spermatogenesis. MP and LD were administered in two separate groups of these animals starting 15^th^ day for a period of 56 days, and the results were compared with an auto-recovery (AR) group. Sperm count and motility, testis histo-architecture, level of reactive oxygen species (ROS), mitochondrial membrane potential (MMP), apoptosis, peripheral hormone levels and testicular germ cell populations were analysed, in all experimental groups. We observed efficient and quick recovery of spermatogenesis in MP and LD groups in comparison to the auto-recovery group. The treatment regulated ROS level, apoptosis, and mitochondrial membrane potential (MMP), recovered the hypothalamic-pituitary-gonadal axis and the number of testicular germ cells, ultimately leading to increased sperm count and motility.

**Conclusion/Significance:**

*M. pruriens* efficiently recovers the spermatogenic loss induced due to EE administration. The recovery is mediated by reduction in ROS level, restoration of MMP, regulation of apoptosis and eventual increase in the number of germ cells and regulation of apoptosis. The present study simplified the complexity of mechanism involved and provided meaningful insights into MP/LD mediated correction of spermatogenic impairment caused by estrogens exposure. This is the first study demonstrating that L-DOPA largely accounts for pro-spermatogenic properties of *M. pruriens*. The manuscript bears CDRI communication number 8374.

## Introduction

Spermatogenic failure in men possibly involves several contributors such as age, oxidative stress, life style, pathological complications, nutritional deficiency, toxicity, exposure to endocrine disruptors such as estrogens etc. which could compromise fertility by affecting sperm count, sperm motility, semen volume and penile erection [1]. Identification of a particular cause and effect relationship in each affected individual is not possible; therefore, a large number of these individuals are labelled as idiopathic. Highly directed therapies such as hormonal intervention have shown poor success [2]. This forces a large number of subjects to choose expensive treatment options such as *in vitro* methods, which a large section of the infertile population fails to afford. Ancient Indian medicinal literature, Ayurveda, cites the use of a large number of plant products by men when the contemporary methods of treatment were not common/available. The use of specific plant products is well documented and has undergone extensive subjective experimentation by the common men, but this lacks scientific evidence supporting the claimed role of these natural products. According to the traditional medicinal system, India is a hub of more than 6000 medicinal plants and about 3000 of them are officially recognized [3].

*Mucuna pruriens* (MP) is a tropical legume known as velvet bean, cowitch and by other common names. The plant originally came from Eastern India and Southern China, where it was at one time widely cultivated as a green vegetable crop [4]. *M. pruriens* has been reported to possess anti-diabetic, anti-neoplastic, anti-microbial, aphrodisiac, and learning and memory enhancing properties [5]. Pro-male fertility properties of MP are supported by few studies including one of our studies on human subjects [4]–[10]. The exact mechanism of its action remains elusive, but possibly it is the result of its anti-oxidant, adaptogenic and general nutritional properties [7]–[9]. Reactive oxygen species (ROS) is now well established to regulate normal sperm function; however, over-production of ROS may result in oxidative stress causing significant adverse impact on semen quality and male fertility [10], [11]. *M. pruriens* seed powder and seed extract both have been reported to be effective in combating the stress mediated compromise in spermatogenesis by maintaining the antioxidant level [6]–[10]. MP is a rich source of L-DOPA and variety of alkaloids, fatty acids, amino acids, minerals and several nutritional elements [11]–[14]. Looking at the promising pro-male fertility and aphrodisiac properties of *M. pruriens*, we undertook the present study to evaluate its potential in recovering spermatogenic loss, and mechanism of its action by study of reactive oxygen species, mitochondrial membrane potential, germ cell apoptosis and DNA content of testicular germ cells.

## Materials and Methods

### Experimental Material

The study was approved by the Institutional Animal Ethical Committee (IAEC) and all experiments involving animals were carried out in strict accordance with the institutional guidelines on the care and use of experimental animals. *M. pruriens* seeds were purchased from an authentic source and identified by the Botany Department of the CSIR-CDRI. Ripened seeds in the month of March were purchased for the whole batch of experiments. The seeds were crushed to powder for experimental purpose. L-DOPA, the major chemical constituents of MP, was purchased from Cayman Chemical (Ann Arbor, Michigan, USA). Annexin V-FITC apoptosis detection kit, 40 µm cell strainer and 5 ml falcon tubes were purchased from BD Biosciences (Franklin Lakes, NJ, USA). Ethinyl estradiol, 2′, 7′-Dichlorofluorescin diacetate, JC-1, RNase A, Propidium iodide and other chemicals were purchased from Sigma-Aldrich (St. Louis, MO, USA).

### Animal Model

We selected ethinyl estradiol to generate the animal model due to wide spread use of estrogens, to which humans are exposed every day. Several different doses of this estrogen were tried to arrive at the selected dosage. Literature suggests minor effect of 1 mg/kg BW/day of EE administration on spermatogenesis, but highly significant effect at a dose of 10 mg/kg BW/day [15]. In another study, histopathological analysis reported a mild negative change in testis and epididymis at a dose of 3 mg/kg BW/day for 2 weeks [16]. We evaluated the impact of EE administration at different doses between 1–10 mg/Kg BW/day for two weeks to arrive at a dose significantly compromising spermatogenesis. We chose 3 mg/kg BW/day of EE administration for a period of 14 days to generate the animal model used in this study. The detailed information and data on development of this model is provided in Table S1 and Figure S1.

### Experimental Design

Male Sprague Dawley (SD) rats (10 Weeks) were maintained at the National Laboratory Animal Centre (NLAC), Central Drug Research Institute, Lucknow, UP, India. The animals were fed standard pellet and water *ad libitum* and all experimental interventions were given orally. Male SD rats were randomly divided into 5 groups. The Group I (sham) represented control animals (N = 14) receiving 0.5% carboxymethylcellulose (CMC), group II (N = 7) III (N = 21) IV (N = 21) and V (N = 21) were administered EE (in 0.5% CMC) at 3 mg/kg BW/day for 14 days to compromise spermatogenesis [16]. After 14^th^ day, group II and 7 animals from group I were sacrificed. Group III was left for auto-recovery for 56 days, receiving only 0.5% CMC during this period. Group IV and V were administered 300 mg/kg BW *M. pruriens* (in 0.5% CMC) and 20 mg/kg BW of L-DOPA (in 0.5% CMC), respectively, for 56 consecutive days on daily basis. The above dose of L-DOPA was calculated assuming approximately 7% L-DOPA content in Indian species of *M. pruriens* [17]. The dosages used in this study were selected after thorough literature review and experimental standardization on animal model in our laboratory (Table S2 and Figure S2). Rest of the animals from group I were used to obtain sperm for use as a positive control, whenever required. Sperm preparation incubated in 100 mM hydrogen peroxide (30%) for five minutes were used as positive controls for ROS and MMP assays. Sperm Count, motility and parameters of specific interest, such as ROS and MMP of sperm, hormone level, testicular apoptosis and testicular germ cell cycle analysis were done in all the groups under investigation. Sperm count, motility and hormone profiling were done at 28, 42 and 56 day post-treatment to see the course of recovery.

### Sperm Count

Sperm count was carried out according to the procedure described by Atessahin et al. [18]. Sperm were collected from the whole epididymis by fine mincing of caudal and caput epididymis in 5 ml of pre-warmed (35°C) PBS. Total number of sperm was determined using Meckler’s sperm counting chamber under light microscope. Approximately 10 µl of diluted sperm suspension was transferred to Meckler’s chamber for counting followed by calculations considering the dilution factor.

### Sperm Motility

Sperm motility parameters were analyzed using the Computer Assisted Semen Analyzer (CASA; HTM-IVOS, Hamilton Thorne, Inc. Beverly, MA, USA). Distal cauda of epididymis was cut and sperm were allowed to leak into 2 ml of M199 medium and maintained at 35°C for 5 minutes so that sperm could diffuse completely in the media. This preparation was diluted 10 times and the sample was measured using a Hamilton Thorne Integrated Visual Optical System (HTM-IVOS) semen analyzer. A total of approximately 200 sperm were analyzed in each sample for calculation of percent motility and percent progressive motility.

### Hormone Profiling

Serum levels of testosterone (T), luteinizing hormone (LH) and follicle stimulating hormone (FSH) were measured by Enzyme-Linked Immunosorbant Assay (ELISA) as per the manufacturer’s instructions. Testosterone ELISA kit was purchased from Calbiotech Inc (Spring Valley, CA, USA). LH and FSH kits were purchased from BMassay (Kaicheng, Beijing, China).

### Histological Architecture

Testicular tissues for histological examination were fixed in 10% formalin for 48 hours and dehydrated by increasing concentrations of isopropanol. After sectioning with microtome, tissues were stained with haematoxylin and eosin. These specimens were examined under a light microscope and digital images were captured at 100×magnification.

### Flow Cytometric Analysis of Testicular Cell Population

Testicular germ cells of the experimental animals were isolated for flow cytometry by the method of Iida et al. [19]. Briefly, the left testis from each animal was excised, decapsulated and placed in cold phosphate buffered saline (PBS) (pH 7.4) containing sodium pyruvate and glucose, followed by gentle mincing with scissor. The cell suspension was pelleted at 400×g for 5 minutes, the supernatant obtained was incubated in 0.05% collagenase in PBS containing 1 mM CaCl_2_ for 60 minutes in a shaking water bath at 37°C. The cell suspension was filtered through a 40 µm BD cell strainer to remove cell aggregates followed by centrifugation at 400×g for 5 minutes. The pellet was washed twice with PBS and resuspended in 10 ml PBS. The cells were fixed in cold 70% ethanol and kept overnight at −20°C, then washed twice in PBS and incubated in 0.1% ribonuclease A solution in PBS at 37°C for 30 minutes. The cells were washed once in PBS and incubated in 0.1% pepsin solution in 0.2% HCl (pH 2.0) at 37°C for 15 minutes. After washing twice with PBS, cells were stained with propidium iodide solution (50 µg/ml in PBS), placed in dark for 30 minutes at 4°C and finally filtered through 40 µm BD cell strainer to remove cell aggregates. Enumeration of cell population on the basis of their DNA content was carried out in a flow cytometer (Model FACS Calibur, BD, USA) at excitation 488 nm and the emission at 580±30 nm.

### Apoptosis Analysis by FACS

Testicular germ cells from control and treated animals were isolated for flow cytometry by the method of Iida et al. as detailed above. After washing with PBS, the cells were re-suspended in 1X binding buffer at a concentration of 1×10^6^ cells/ml and then 100 µl of suspension was transferred to 5 ml BD falcon. BD FITC - Annexin V apoptosis detection kit was used as per manufacturer’s protocol. Briefly, 5 µl of FITC Annexin V and 5 µl of PI were added to the suspension and cells were gently vortexed and incubated for 15 minutes at room temperature (25°C) in the dark. 400 µl of 1X binding buffer was added to each tube followed by analysis on FACS calibur. Enumeration of cells on the basis of their DNA content was carried out in a flow cytometer (Model FACS Calibur, BD, USA) at excitation wavelength of 488 nm and the emission at 580±30 nm. Fluorescence compensation was done using 4-tube protocol in which the tubes with unstained cells, Annexin V-FITC only, PI only and AnnexinV−FITC+PI were used for initial settings, before analyzing control and treatment groups. Cluster of events located in lower left quadrant (AnnexinV −, PI −) are viable cells, in lower right quadrant (Annexin V+, PI−) are apoptotic, in upper right are necrotic (Annexin V +, PI +) and in upper left are dead cells (AnnexinV−, PI +).

### Measurement of Mitochondrial Membrane Potential (MMP)

JC-1 (5, 5′, 6, 6′ tetrachloro–1, 1′, 3, 3′–tetraethylbenzimadazolylcarbocyanine iodide), a cationic dye, was used for MMP measurement. Sperm from caudal portion of epididymis were collected in 2 ml PBS and counted on Meckler’s chamber followed by dilution to a concentration of ∼ 5 × 10^6^ million sperm/ml while maintaining the suspension at 35°C in an incubator. 500 µl of sperm suspension was taken in BD falcon after filtering through 40 µm BD cell strainer to remove unwanted cell debris, if any, followed by staining with 1 µl of 1.53 mM JC-1 for 40 minutes at 37°C. 500 µl sperm suspension with H_2_O_2_ added at a final concentration of 100 mM incubated for 5 minutes before JC-1 staining was used as a positive control (PC). Samples were analyzed by BD FACS calibur for a minimum of 10,000 cells to measure JC-1 fluorescence at excitation wavelength of 488 nm and emission at 529 nm and 590 nm for J-monomeric (green fluorescence) and J-aggregate (red fluorescence) forms, respectively, with a flow rate of 400–450 cells/second. The ratio of red fluorescence (R1) to green fluorescence (R2) was calculated to get the mitochondrial membrane potential. Higher value of the ratio indicates better mitochondrial functioning.

### Determination of ROS by Flow Cytometry

Intracellular ROS in sperm cells was measured using the fluorescent probe DCFH-DA (2′, 7′-dichlorofluorescin diacetate). Cauda was cut at the tip for releasing sperm in the PBS followed by filtration through 40 µm BD filter. 500 µl of sperm suspension was taken, maintaining ∼ 5×10^6^ cells and DCFH-DA was added to a final concentration of 2.5 µM. 500 µl sperm suspension with H_2_O_2_ added at a final concentration of 100 mM after incubating for 5 minutes, before DCFH-DA addition was used as a positive control. DCFH-DA loaded sperm cells were incubated for 30 minutes at 35°C. The fluorescence intensity of 2′, 7′-dichlorofluorescein (DCF) in cells was analyzed fluorometrically at 340 and 525 nm for excitation and emission, respectively, with a flow rate of 300–400 cells/sec for minimum 8,000 cells. DCFH-DA conversion into DCF was recorded for the estimation of ROS in sperm cells.

### Statistical Analysis

Statistical comparison of the data was done using Student’s ‘t’ test or ANOVA and P values <0.05 were considered to be significant. To measure the effect of EE on various parameters, the values for EE group were compared with the control group using Student’s ‘t’ test. In order to measure the efficacy of MP and LD in restoring various parameters, data between MP, LD and AR groups were compared using ANOVA. Results were presented as Mean±SD.

## Results

### Sperm Count and Motility

Treatment with EE for 14 days compromised sperm count, percent sperm motility and percent progressive motility, providing us a suitable model for testing efficacy of MP in recovering spermatogenic loss (Table 1). Animals left for auto-recovery could regain sperm count and motility to a significant extent after 56 days. In contrast to AR, treatment with MP helped a highly significant and fast recovery of both sperm count and motility. The level of recovery in MP group was higher in comparison to the AR group at all time points. The values after treatment were not only significantly higher in comparison to AR, but sometimes surpassed the normal mean values seen in the control group. Similar treatment with LD also helped better recovery of all the three sperm parameters in comparison to the AR group; however, the values reached statistical significance only in the case of sperm count (Table 1). Apart from a faster recovery of spermatogenesis, MP and LD also resulted in better sperm parameters at the end of the treatment period.

**Table 1:** A time-line profile of sperm count and motility during treatment.

Sperm parameter	Days	Control	EE	AR	MP	LD
Sperm count (million/ml)	14	202.1±15.4&	26.1±4.3***			
	28			43.5±4.5	61.1±6.9***	58.6±3.7***
	42			100.5±4.32	127.3±4.32***	118.5±4.5***
	56			174.2±8.4	214.6±11.9***	205.5±7.2***
% Sperm motility	14	60.5±5.1	16.8±9.0***			
	28			23.3±0.98	33.0±1.52***	28±0.93*
	42			31.5±3.39	46.1±3.12***	36.0±4.38
	56			49.3±7.8	64.3±3.6**	61.83±4.1**
% Progressive motility	14	17.5±2.0	4.5±2.0***			
	28			7.5±0.54	8.83±1.16	8.5±1.37
	42			9.0±1.41	12.1±1.72*	11.0±1.41
	56			16.6±4.0	21.67±5.7	18.83±2.4

For statistical inference, EE was compared with control group, and MP and LD were compared with AR group.

& the values (mean±SD) are average of data for 6 animals. Statistical significance is indicated as * P<0.05, ** P<0.005, *** P<0.0005.

### Serum Hormone Levels

EE administration significantly reduced the serum testosterone, FSH and LH levels in comparison to the control group (Table 2). Treatment with MP and LD resulted in significant recovery of the endocrine axis as compared to the AR group. Both MP and LD showed recovery of the endocrine axis at all time points, but the differences were more significant in the case of MP. At the end of the treatment period, the levels of all three hormones were higher than the control group (Table 2). This suggests action of *M. pruriens* at the central nervous system.

**Table 2:** A time-line profile of the hormone levels during treatment**.**

Hormone	Days	Control	EE	AR	MP	LD
Testosterone (ng/ml)	14	1.25±0.12&	0.19±0.07***			
	28			0.38±0.04	0.61±±0.08***	0.50±0.07*
	42			0.73±0.08	1.03±0.18**	0.85±0.10
	56			1.13±0.09	3.05±0.91*	1.60±0.22
FSH (mIU/ml)	14	5.91±0.58	1.22±0.30***			
	28			1.90±0.5	2.90±0.51*	2.60±0.49
	42			3.47±0.56	4.07±0.46	3.77±0.75
	56			5.83±0.42	6.15±0.44	5.86±0.33
LH (mIU/ml)	14	3.65±0.46	1.15±0.15			
	28			1.66±0.32	2.17±0.42	1.80±0.3
	42			2.13±0.1	3.26±0.55**	2.61±0.47
	56			3.49±0.48	4.18±0.71	4.09±0.81

For statistical inference, EE was compared with control group, and MP and LD were compared with AR group.

& the values (mean±SD) are average of data for 6 animals. Statistical significance is indicated as * P<0.05, ** P<0.005, *** P<0.0005.

### Histological Architecture

**Figure 1:**
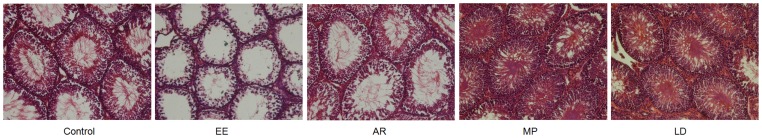
Histological architecture of the testis in different treatment groups. Control group showed normal testicular histology with the lumen of seminiferous tubules filled with sperm, EE group showed significant compromise in spermatogenesis with almost empty lumens and degeneration of Sertoli cells. AR group showed poor recovery in comparison to complete recovery in MP and LD groups evidenced by densely filled seminiferous tubules and healthy Sertoli cells attached to the basement membrane.

Animals receiving EE administration exhibited differences in the testicular histology, particularly the number and arrangement of the Sertoli and spermatogenic cells in the seminiferous tubules (Figure 1). The lumina of seminiferous tubules were particularly enlarged with Sertoli cells shrinking towards the basement membrane coupled with significant reduction in the number of spermatids in the lumen. In AR group, the Sertoli cells regained their normal architecture and the lumina were occupied by the spermatids. Treatment with MP and LD resulted in much better recovery of the luminal architecture, with the Sertoli cells regaining their normal position and the spermatids completely filling the lumina (Figure 1).

### ROS Level Before and After Treatment

**Figure 2:**
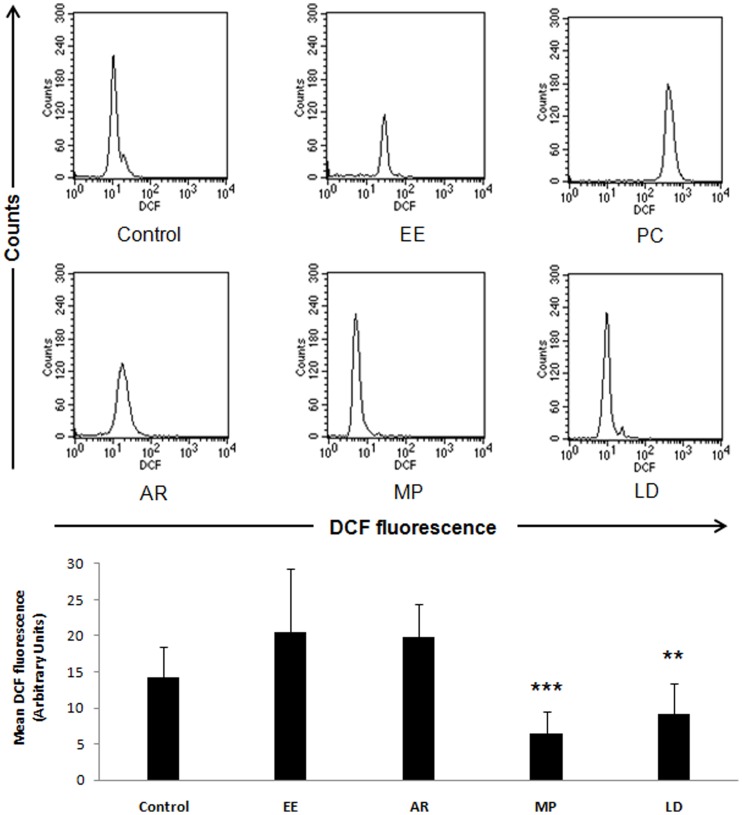
Comparison across the treatment groups showed significant increase in ROS upon ethinyl estradiol administration and in the positive control group. AR group showed slight improvement, but highly significant recovery was seen in the MP and LD groups. Bar diagram shows quantitative assessment of ROS (mean DCF fluorescence). Positive control group was not shown in the bar diagram due to very high ROS value in this group. Data are expressed as Mean ± SD (n = 6). Statistical significance is indicated as **P<0.005, ***P<0.0005 vs. AR.

Ethinyl estradiol administration increased the ROS level significantly (Figure 2). In EE treated group, very small sperm population could be retrieved from the caudal epididymis due to apoptotic death of sperm, possibly resulting in debris formation inside the epididymis. The loss in the sperm population (decreased vertical scale) and increase in ROS level (right shift in horizontal scale) is evident in the FACS analysis (Figure 2). Natural disposal of ROS was only little as seen in the AR group. On the other hand, treatment with MP and LD helped combat ROS and recover the number of sperm in swim up, making the differences statistically significant (Figure 2).

### Mitochondrial Membrane Potential Before and After Treatment

**Figure 3:**
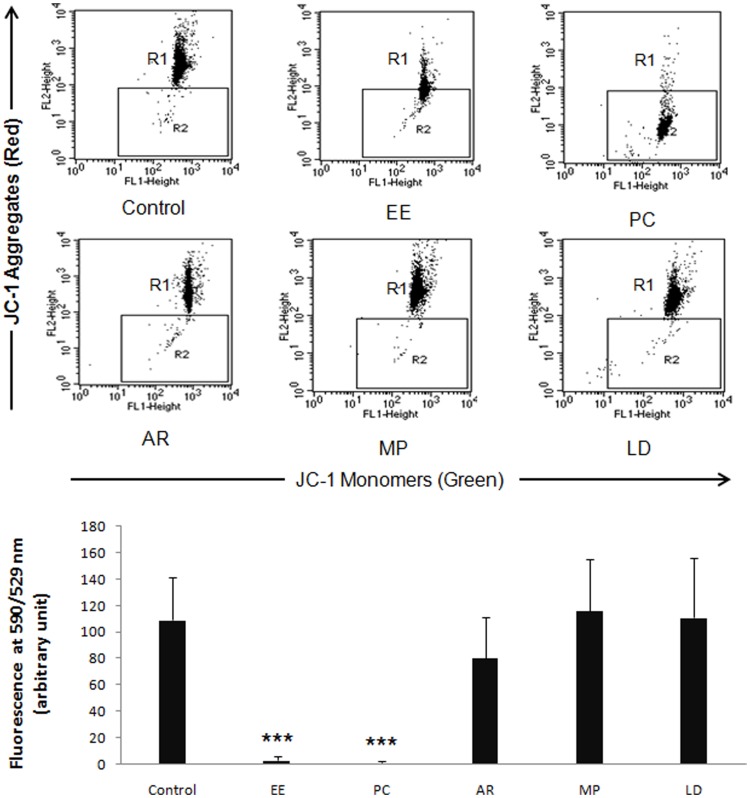
EE and positive control groups showed significant loss of MMP. AR group showed recovery of MMP, but a higher level of restoration was seen in the MP and LD groups. The bar diagram depicts the quantitative assessment of MMP across the treatment groups. Data are expressed as Mean ± SD (n = 6). Statistical significance is indicated as ***P<0.0005 vs. control.

Ethinyl estradiol administration significantly reduced MMP (shift of the cell cloud from R1 to R2), showing the damage caused to the sperm mitochondria (Figure 3). AR group recovered the loss of MMP to a significant extent. MP was distinguishable by significant impact on MMP improvement. Similarly, the recovery potential of LD was comparable (Figure 3).

### Quadrant Analysis of Apoptosis

**Figure 4:**
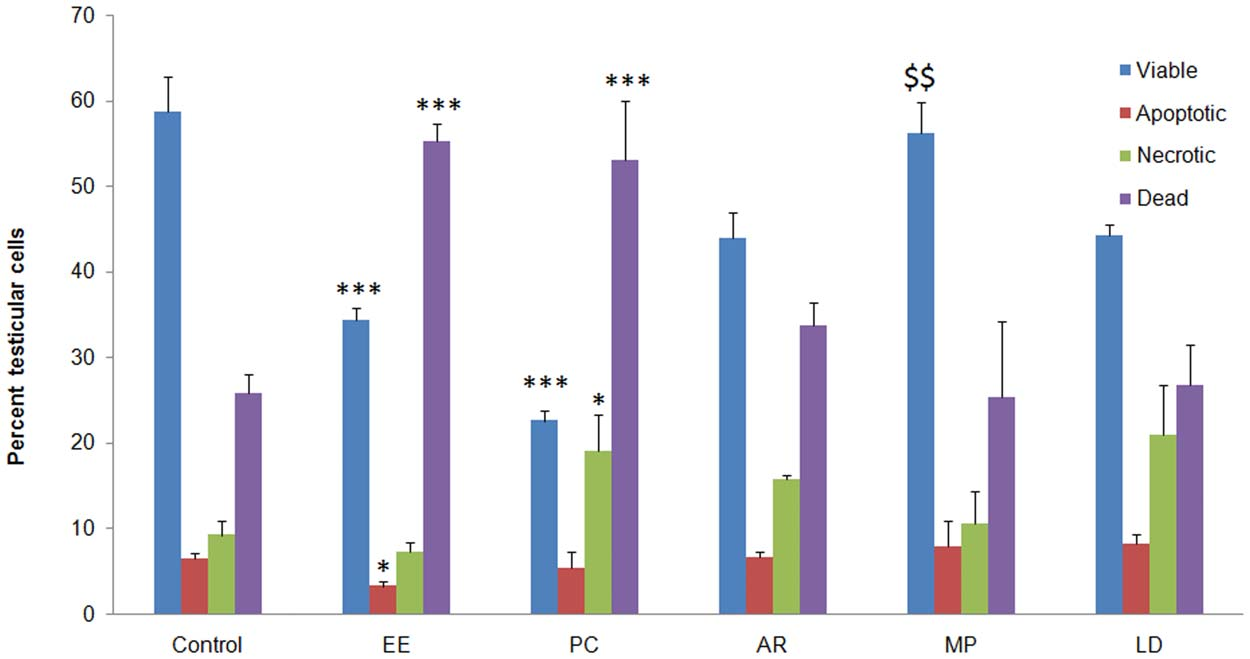
Bar diagram comparing the level of apoptosis across the treatment groups. Ethniyl estradiol administration promoted germ cell apoptosis, resulting in significant increase in dead cells and a corresponding decreased in viable, apoptotic and necrotic cells. Restoration of testicular cells with different fates was seen in the auto-recovery group. MP appeared to be the best in restoring the balance between cell types, but a relatively lesser effect was observed in the LD group. Data are expressed as Mean ± SD (n = 6). Statistical significance is indicated as *P<0.05, **P<0.005, ***P<0.0005 vs. control and ^$$^ P<0.005 vs. AR.

FACS analysis showed that the level of apoptosis was significantly higher in the EE and PC groups as compared to control (Figure 4). Impact of treatment in this experiment could be understood by looking at reduction in the number of necrotic cells against an increase in apoptotic cells falling in other compartments of the apoptotic quadrant. EE administration decreased the number of viable, early apoptotic and late apoptotic cells and increased the number of necrotic cells. This decrease could indicate conversion of apoptotic cells into necrotic or promotion of direct cell death as a result of this treatment. This trend in cell death was confirmed by the positive control. Animals left for auto-recovery showed lesser number of necrotic cells, and apoptotic cell number comparable to the EE group. Cell death was significantly checked by treatment with MP and LD (Figure 4). The values in the four quadrants of MP group were comparable to the control group, which could not be achieved by auto-recovery.

### DNA Content Analysis of Testicular Cells

**Figure 5:**
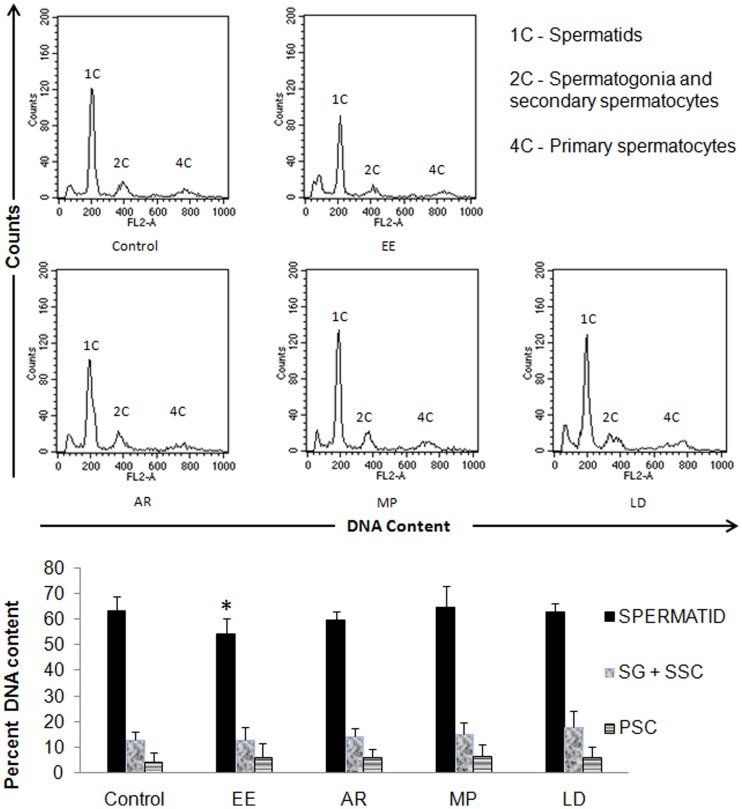
DNA content analysis of the testicular cell population in the treatment groups. A significant reduction in spermatid population (1C) after ethinyl estradiol treatment was observed, which was recovered to certain extent in the auto-recovery group. Significant recovery of spermatid population was seen in the MP and LD groups. Data are expressed as Mean ± SD (n = 6). Statistical significance is indicated as *P<0.05 vs. control. SG = Spermatogonia, SSC = Secondary Spermatocytes, PSC = Primary Spermatocytes.

Testicular cells with four sub-populations were captured, but we considered three relevant peaks of our interest for data analysis (Figure 5). EE administration significantly reduced the number of spermatids with an eventual increase in the number of primary spermatocytes without any alteration in the population of secondary spermatocytes (Figure 5). In the auto-recovery group, mild recovery of spermatid population was seen without much change in the other cell populations. Treatment with MP and LD helped achieve complete recovery, restoring back the proportion of spermatids with mild increase in the number of primary and secondary spermatocytes including spermatogonia (Figure 5).

## Discussion

Ethinyl estradiol induced imbalance between apoptosis and cell proliferation, and sex steroid disruption in a testis culture of gudgeon, *Gobio gobio*, altered reproductive behaviour, circulating hormones particularly reduced testosterone, and sexual morphology in male fathead minnows [20], increased ROS, germ cell apoptosis, exfoliation of germ cells from the seminiferous epithelium accompanied with spermiation failure and depletion in serum testosterone, LH and FSH level in rats [21], [22], [23]. Therefore, exposure to EE may compromise spermatogenesis, male fertility and even male sexual differentiation if exposed in early stages of development. Ethinyl estradiol administration in rats for 14 days suppressed hypothalamic-pituitary-gonadal endocrine axis (Table 2), induced oxidative stress causing germ cell apoptosis (Figure 4), decreased mitochondrial membrane potential, resulting in significant reduction in sperm count and motility (Table 1). It is worth noting that the physiological disturbances caused by ethinyl estradiol administration match the anomalies seen in a large number of infertile human individuals, justifying the selection of this animal model.

Research over the last one decade has identified oxidative stress as a significant contributor of male infertility [24], [25]. Other complicating factors such as deficiency of certain co-factors and essential minerals could further elevate the impact of damage [26], [27]. Oxidative stress has a direct adverse effect on sperm count and motility as excess of ROS induces germ cell apoptosis and loss of mitochondrial membrane potential [28]. A direct relationship between sperm motility and activity of respiratory chain enzymes of mitochondria has been reported, reflecting important role of mitochondria in sperm motility [29]. Upon *M. pruriens* administration, we observed significant recovery of mitochondrial membrane potential coupled with a gain in sperm motility (Figure 3). The relationship between ROS and MMP, and between MMP and sperm motility has been reported in different species including humans [30], [31]. Infertility in humans presents a complex picture of disturbances at the hormone level, generation and disposal of ROS and the actual process of spermatogenesis. *M. pruriens* affected all these parameters, ultimately helping recover both the quality and quantity of sperm production (Table 1). The complex etiological nature of male infertility demands an equally complex and multi-pronged therapy. This may be one of the reasons behind poor success of highly directed therapies such as hormonal intervention and excellent performance of *M. pruriens*. This could be attributed to its nutritional, adaptogenic, aphrodisiac and restorative properties. *M. pruriens*, thus, has the potential to treat compromised fertility, characterized by loss of sperm count and motility, and perhaps provide immunity against such complications. This property of *M. pruriens* has been supported by our previous study on infertile men [6]–[10].

The most interesting finding of our study is deciphering the possible mechanism of action of *M. pruriens*. We found that the major effect of *M. pruriens* on spermatogenesis was mediated by recovery of endocrine axis suppressed as a result of EE administration (Table 2). *M. pruriens* helped an early recovery of the endocrine axis and spermatogenesis. At the end of the treatment period, the sperm count and motility exceeded the normal mean values, which could be due to elevated hormone levels, promoting the process of spermatogenesis, and/or due to reduced number of sperm elimination during quality control in the epididymis as a result of qualitatively better sperm production [32]. We extended the investigation to measure effect on other parameters affecting spermatogenesis such as increased ROS level, loss of mitochondrial membrane potential, testicular cell death and germ cell development. Interestingly, *M. pruriens* helped normalize ROS level and MMP efficiently in comparison to the auto-recovery group (Figures 2 and 3). Measuring mitochondrial membrane potential is a very sensitive test and is considered to be the best parameter to analyze sperm quality during IVF procedure in human [33]. Loss of mitochondrial membrane potential upon ethinyl estradiol treatment and its efficient recovery upon *M. pruriens* administration suggests that MP is effective in improving mitochondrial health and so the sperm motility.

*M. pruriens* was also efficient in restoring the balance between viable, apoptotic, necrotic and dead cells (Figure 4). Our observation of improved testicular germ cells viability in *M. pruriens* treated group may be due to elevated level of FSH or testosterone or their synergistic effect, preventing germ cell apoptosis and promoting viability [34]. This could also explain increase in sperm count beyond the normal mean values at the end of the treatment. Testicular cell population analyzed before and after treatment showed actual increase in the number of sperm cells with a concomitant increase in other testicular cell populations, which was supported by testicular sectioning (Figure 1). From the above, it is worth noting that *M. pruriens* combats compromised spermatogenesis by targeting the physiological anomalies accused to be the most common cause of infertility in humans. Since *M. pruriens* is a rich source of L-DOPA, we proposed to evaluate if pro-fertility activity of MP could be largely attributed to its L-DOPA content. Experiments using L-DOPA showed efficient recovery of endocrine axis, spermatogenesis and other biochemical parameters in comparison to the auto-recovery group. L-DOPA was not as efficient as *M. pruriens*, but comparable effects suggest that L-DOPA is one of the major active constituents of *M. pruriens*. *In vitro* antioxidant assays have supported antioxidant property of L-DOPA [35]. Dopamine, a product of L-DOPA metabolism, was also found to possess strong anti-oxidant capacity and free radical scavenging activity [36], [37]. L-DOPA probably stimulates the hypothalamus and forebrain, thus stimulating secretion of gonadotropin releasing hormone (GnRH), which ultimately activates anterior lobe of pituitary to secrete FSH and LH. Elevated levels of FSH and LH stimulate the process of spermatogenesis via testosterone.

Environmental estrogens mimicking estradiol are ubiquitous and humans are exposed to them daily by a number of routes [38]. Estrogenic effects are not restricted to a small group of therapeutic agents, but appear in several groups of compounds that are used daily in industry, agriculture or in homes [39]. Such exposure could severely affect spermatogenesis and effects could be irreversible or transmitted to the subsequent generations in case of exposure in neonatal stage [38]–[40]. Promising recovery of spermatogenesis upon *M. pruriens* administration after ethinyl estradiol induced loss of sperm count and motility suggests its use in alleviating the adverse effects of environmental exposure to estrogens. The Indian Ayurvedic system recommends the use of such herbs as protective health supplements to strengthen the natural defence against health hazards. Therefore, this plant could be further explored as a pro-health supplement in general, apart from its use in treatment of reproductive ailments such as infertility.

In conclusion, *M. pruriens* has the potential to recover spermatogenic loss, which makes it the treatment of choice not only for infertile individuals, but also for extraction of quality sperm for use in *in vitro* fertilization procedures. Exposure to health hazards such as environmental estrogens, affects spermatogenesis, which is evident by declining semen quality over the last few decades [41]. Reversal of the estrogen mediated loss of testicular homeostasis and spermatogenesis confers an added advantage to the use of this plant material. The pro-health effects of this plant could be due to the presence of a complex mixture of alkaloids and its nutritional, anti-oxidant and adaptogenic properties. The presence of L-DOPA accounts for majority of the activity; however, other yet to be identified important constituents are likely to contribute to the overall activity. Looking at our findings on animal model and infertile human subjects, we suggest that *M. pruriens* improves spermatogenesis by recovery of the endocrine axis and testicular homeostasis, leading to better semen quality. Further research on this plant could explore signalling pathway associated with the end effect. We believe that the data presented in this article are really encouraging and identify the first lead towards understanding the mechanism of action of this wonderful medicinal plant.

## Supporting Information

Figure S1 **Upper panel: A highly significant dose dependent reduction in sperm count was observed at all the doses.** Middle panel: A highly significant reduction in sperm motility was observed at doses 3 mg and 6 mg/kg BW/day, while at 9 mg/kg BW/day no motility was seen. Lower panel: The number of progressively motile sperm was significantly reduced at 3 mg/kg BW/day, while no progressive motility was seen at doses of 6 and 9 mg/kg BW/day. Data are expressed as Mean ± SD (n = 6). Statistical significance is indicated as ***P<0.0005 vs. control.
(TIF)

Figure S2 **Different doses of** ***M. pruriens*** **were tried for recovery of sperm count and motility.** The best response was seen at a dose of 300 mg/Kg body weight per day, which was selected as the experimental dose for treatment trails. Statistical significance is indicated as *P<0.05, **P<0.005, ***P<0.0005 vs. auto-recovery.
(TIF)

Table S1 **Development of the animal model with compromised spermatogenesis.**
(DOC)

Table S2 **Standardization of** ***M. pruriens*** **dosage for recovery of spermatogenic loss.**
(DOCX)
