# Supplementary figures and images for: Mucuna pruriens and Its Major Constituent L-DOPA Recover Spermatogenic Loss by Combating ROS, Loss of Mitochondrial Membrane Potential and Apoptosis
Source: PLoS One. 2013 Jan 22;8(1):e54655. doi: 10.1371/journal.pone.0054655 (PMC3551850; doi:10.1371/journal.pone.0054655)

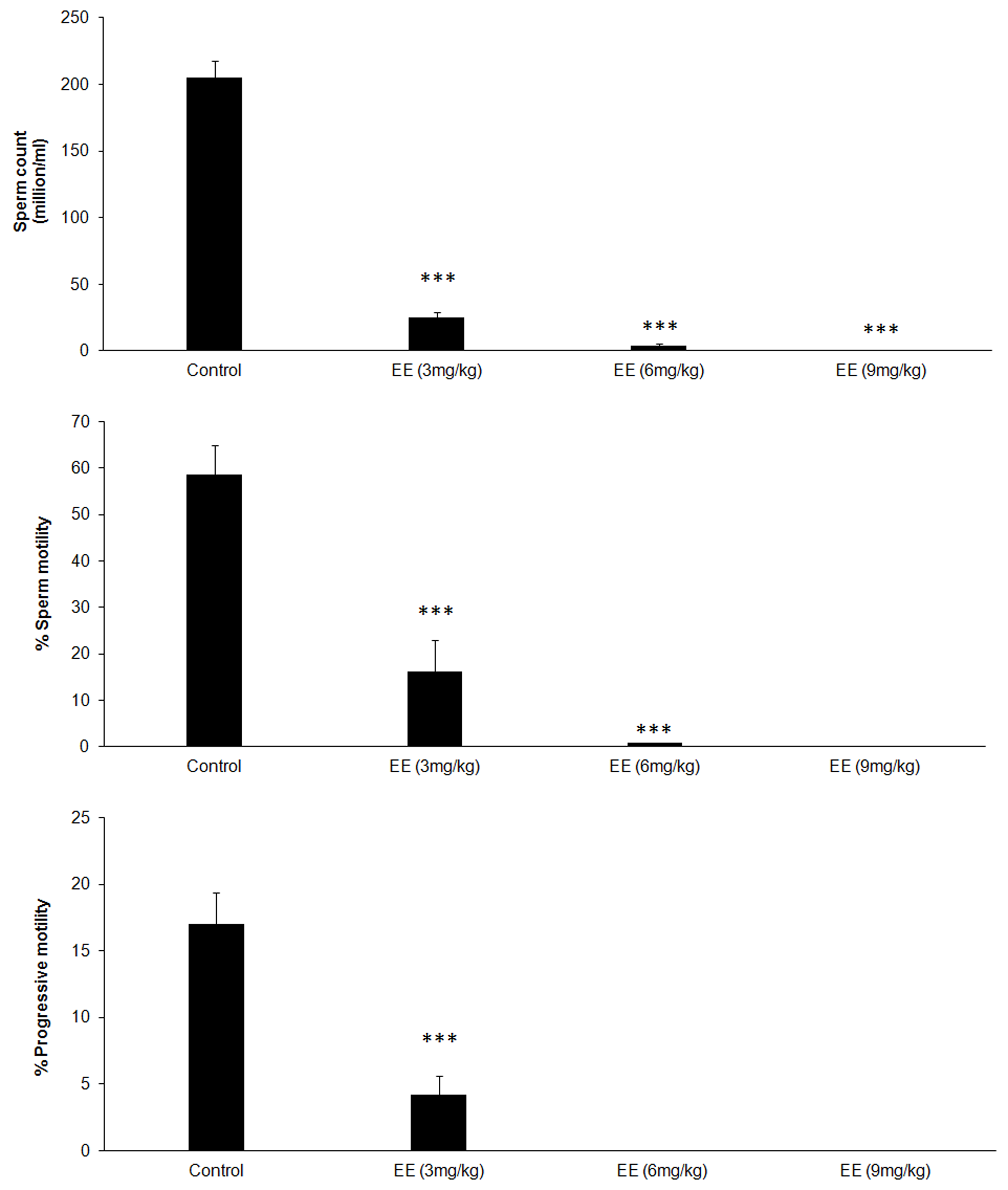

Supplement: Figure S1 — Upper panel: A highly significant dose dependent reduction in sperm count was observed at all the doses. Middle panel: A highly significant reduction in sperm motility was observed at doses 3 mg and 6 mg/kg BW/day, while at 9 mg/kg BW/day no motility was seen. Lower panel: The number of progressively motile sperm was significantly reduced at 3 mg/kg BW/day, while no progressive motility was seen at doses of 6 and 9 mg/kg BW/day. Data are expressed as Mean ± SD (n = 6). Statistical significance is indicated as ***P<0.0005 vs. control. (TIF) [file pone.0054655.s001.tif]

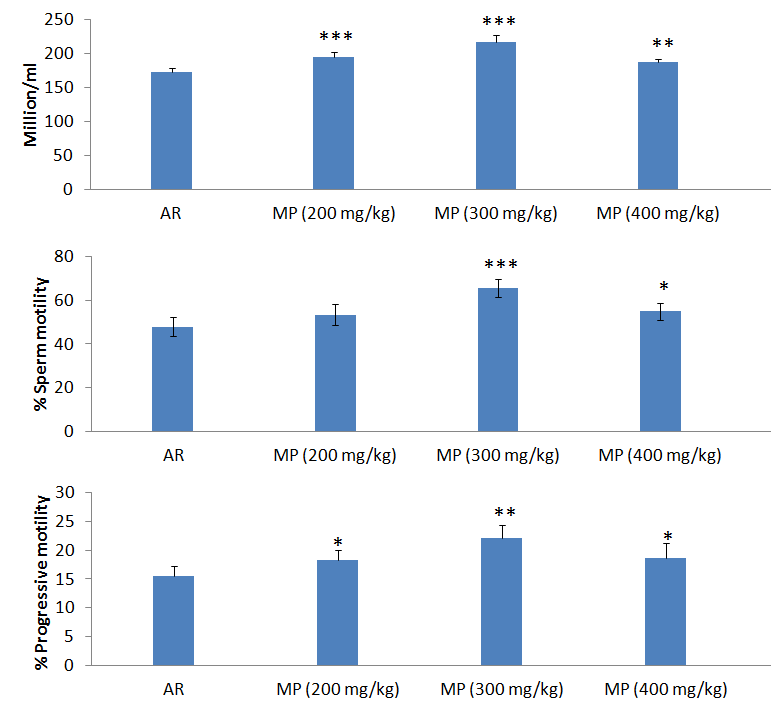

Supplement: Figure S2 — Different doses of M. pruriens were tried for recovery of sperm count and motility. The best response was seen at a dose of 300 mg/Kg body weight per day, which was selected as the experimental dose for treatment trails. Statistical significance is indicated as *P<0.05, **P<0.005, ***P<0.0005 vs. auto-recovery. (TIF) [file pone.0054655.s002.tif]
