# Supplementary material for: Mucuna pruriens and Its Major Constituent L-DOPA Recover Spermatogenic Loss by Combating ROS, Loss of Mitochondrial Membrane Potential and Apoptosis
Source: PLoS One. 2013 Jan 22;8(1):e54655. doi: 10.1371/journal.pone.0054655 (PMC3551850; doi:10.1371/journal.pone.0054655)
Supplement: Table S1 — Development of the animal model with compromised spermatogenesis. (DOC) [file pone.0054655.s003.doc]

**Supplementary Table S1**

| Parameters | Control | EE (3mg/kg BW/day) | EE (6 mg/kg BW/day) | EE (9 mg/kg BW/day) |
| --- | --- | --- | --- | --- |
| Sperm count | 205±12.93 | 25.33±3.93*** | 3.67±1.86*** | 0.15±0.10*** |
| % Sperm motility | 58.5±6.41 | 16.17±6.77*** | 0.83±0.75*** | 0.00 |
| % Prog. motility | 17.00±2.37 | 4.17±1.47*** | 0.00 | 0.00 |

*** P < 0.0005

**Foot note:** Previous studies on ethinyl estradiol (EE) treatment and its effects on testis and epididymal sperm helped us develop a model of compromised spermatogenesis (Masako et al, 1999; Toshiko et al, 2000). We didn’t use a very high dose fearing irreversible changes in the testis. Doses of 3 mg, 6 mg and 9 mg/kg BW/day were administered orally and sperm parameters were collected. Sperm count and motility data were gathered for 6 animals per group. A dose of 3 mg/kg BW/day was found suitable to generate animal model with spermatogenesis compromised significantly, but in a reversible manner.

**References**

Masako K, Susumu K, Atsuko H, Kurajiro K (1999) Epididymal sperm motion as a

parameter of male reproductive toxicity:sperm motion, fertility, and histopathology in

ethinylestradiol-treated rats. Reprod Toxicol 13: 279–289.

Toshiko K, Miwa S, shuji O, Ayako I, Akiko M, et al. (2000) Collaborative work to

evaluate toxicity on male reproductive organs by repeated dose studies : Effects of repeated

doses of ethinyl estradiol for 2 and 4 weeks on male reproductive organs. J. Toxicol Sci 25:

43-49
