# Supplementary material for: Mucuna pruriens and Its Major Constituent L-DOPA Recover Spermatogenic Loss by Combating ROS, Loss of Mitochondrial Membrane Potential and Apoptosis
Source: PLoS One. 2013 Jan 22;8(1):e54655. doi: 10.1371/journal.pone.0054655 (PMC3551850; doi:10.1371/journal.pone.0054655)
Supplement: Table S2 — Standardization of M. pruriens dosage for recovery of spermatogenic loss. (DOCX) [file pone.0054655.s004.docx]

**Supplementary Table S2**

| Parameters | Auto-recovery | *M. pruriens*  (200 mg/kg/day) | *M. pruriens*  (300 mg/kg/day) | *M. pruriens*  (400 mg/kg/day) |
| --- | --- | --- | --- | --- |
| Sperm count | 171.83±6.91 | 194.5±6.41*** | 216.6±10.19*** | 187.5±4.23** |
| Sperm motility | 47.8±4.36 | 53.5±4.89 | 66.63±4.22*** | 55.0±3.79* |
| Prog. motility | 15.5±1.76 | 18.33±1.75* | 22.17±2.29** | 18.67±2.58* |

The values (mean + SD) are average of data for 6 animals. Statistical significance is indicated as * P < 0.05, ** P < 0.005, *** P < 0.0005.

**Foot note**: Based on earlier studies demonstrating effects of *M. pruriens* on sperm parameters (Suresh et al, 2010), we tried 200 mg/kg, 300 mg/kg and 400 mg/kg BW/day for treatment of spermatogenic loss and compared it with auto-recovery (no treatment) group after a period of 56 days. The best response was seen using 300 mg/kg, which was selected as experimental dose.

**Reference**

Suresh S, Prithiviraj E, Prakash S (2010) Effect of *Mucuna pruriens* on oxidative stress mediated damage in aged rat sperm. Int J Androl 33: 22-32.
